# Prostate Cancer Detection Rate of Manually Operated and Robot-assisted In-bore Magnetic Resonance Imaging Targeted Biopsy

**DOI:** 10.1016/j.euros.2022.05.002

**Published:** 2022-05-28

**Authors:** Mads Sandahl, Kristian Juul Sandahl, Edvard Marinovskij, Tomas Frahm Nielsen, Karina Dalsgaard Sørensen, Michael Borre, Benedicte Parm Ulhøi, Bodil Ginnerup Pedersen

**Affiliations:** a Department of Radiology, Aarhus University Hospital, Aarhus, Denmark; b Department of Clinical Medicine, Aarhus University, Aarhus, Denmark; c Department of Molecular Medicine (MOMA), Aarhus University Hospital, Aarhus, Denmark; d Department of Urology, Aarhus University Hospital, Aarhus, Denmark; e Department of Pathology, Aarhus University Hospital, Aarhus, Denmark

**Keywords:** Prostate cancer, In bore, Magnetic resonance imaging targeted prostate biopsy, Magnetic resonance imaging targeted biopsy, Biopsy, Manually operated, Robot assisted, Magnetic resonance imaging

## Abstract

**Background:**

The diagnostic efficacy regarding prostate cancer (PC) detection by manually operated in-bore magnetic resonance imaging (MRI) targeted prostate biopsy (MO-MRGB) versus robot-assisted in-bore MRI targeted prostate biopsy (RA-MRGB) is lacking evidence.

**Objective:**

We hypothesized that the detection rates (DRs) for PC of MO-MRGB and RA-MRGB were similar and aimed to compare these.

**Design, setting, and participants:**

We prospectively included all patients who received in-bore MRI targeted prostate biopsy (MRGB) of the prostate in the Central Denmark Region from August 2014 to February 2020. From August 2014, MO-MRGB was used, and from March 2018, RA-MRGB was preferred. Referral to in-bore MRGB was based on multiparametric MRI (mpMRI).

**Outcome measurements and statistical analysis:**

We compared PC DRs of MO-MRGB and RA-MRGB with Pearson’s chi-square test. We made three binary regression models and calculated the risk difference (RD) of PC between the in-bore MRGB systems.

**Results and limitations:**

A total of 3107 patients were referred to mpMRI, and 884 (28%) patients went on to receive in-bore MRGB. The MO-MRGB and RA-MRGB systems were used in 505 (57%) and 379 (43%) patients, respectively. Taking clinically relevant covariates into account, we found no statistically significant difference in PC DRs between MO-MRGB and RA-MRGB (72% vs 73%, RD 1%, 95% confidence interval –4% to 7%, *p* = 0.6). The main limitation was a shift in population characteristics.

**Conclusions:**

We did not see evidence of an effect on the DR or the RD for PC when we compared MO-MRGB with RA-MRGB. Cost effectiveness should be considered carefully when choosing the MRGB system.

**Patient summary:**

We compared two magnetic resonance imaging guided prostate tissue sampling systems regarding prostate cancer (PC) detection. One system was manually operated, and the other system was robot assisted. Comparing the systems, we found no evidence of a difference in their ability to detect PC.

## Take Home Message

We did not see evidence of an effect on the detection rate or the risk difference for prostate cancer between manually operated and robot-assisted in-bore magnetic resonance imaging targeted biopsy. Cost effectiveness should be considered carefully when choosing the biopsy system.

## 1 Introduction

Traditionally, transrectal ultrasound guided (TRUS) biopsy has been the preferred biopsy method when prostate cancer (PC) was suspected [1]. However, multiparametric magnetic resonance imaging (mpMRI) of the prostate has increasingly been used and is now recommended before biopsy by the European Association of Urology [2], [3].

Magnetic resonance imaging (MRI) targeted prostate biopsy (MRGB) is based on MRI, by visualizing the exact lesion of interest, contrary to the systematic sampling with TRUS biopsy [4]. MRGB can be performed as cognitive MRI guided TRUS biopsy, MRI TRUS-fusion biopsy, or MRI in-bore biopsy, all of which have similar detection rates (DRs) for PC [5], [6]. During in-bore MRGB, the patient remains prone in the MRI scanner, while the biopsy is performed transrectally [6].

**Figure 1:**
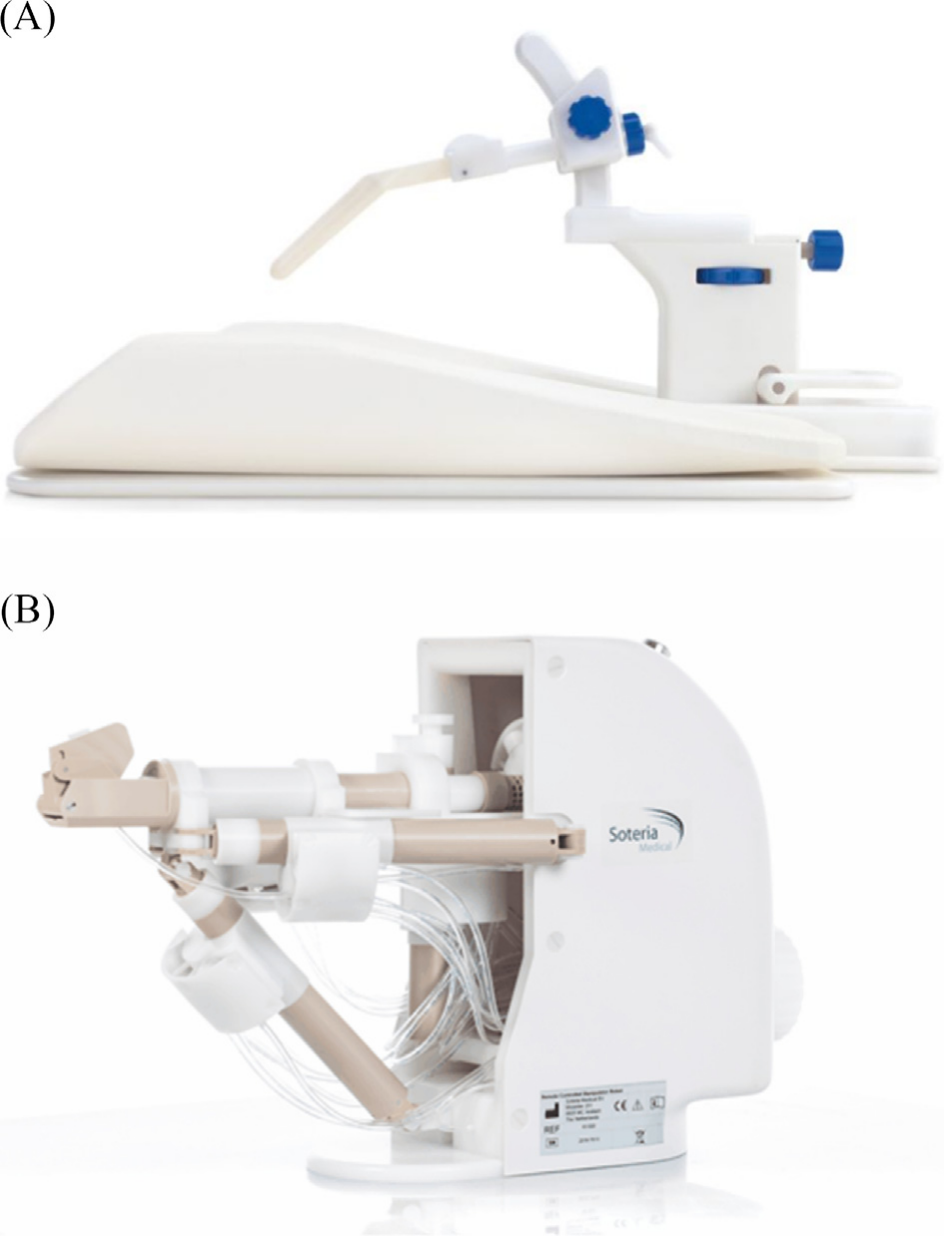
(A) The manually operated in-bore MRGB system (Philips, DynaTRIM; Invivo Corp) and (B) the robot-assisted in-bore MRGB system (Soteria Medical). MRGB = magnetic resonance imaging targeted prostate biopsy.

Initial reports on in-bore MRGB described the method by using in-house constructed systems, but now in-bore MRGB systems are commercially available [7], [8], [9], [10]. In-bore MRGB can be performed with different needle-guide systems as either manually operated in-bore MRGB (MO-MRGB) or robot-assisted in-bore MRGB (RA-MRGB; Fig. 1). In the latter, the radiologist remotely controls a pneumatically actuated robot [7], [11].

The aim of this prospective quality assessment study was to compare MO-MRGB with RA-MRGB. We hypothesized that MO-MRGB performed similar to RA-MRGB, that is, we would find the same PC DR and the risk difference (RD) of PC between the systems was close to zero.

## 2 Patients and methods

### 2.1 Study design

We conducted a single-center, prospective, and consecutive quality assessment study comparing two different in-bore MRGB systems.

Quality assessment is a mandatory part of Danish hospital regulatory guidelines introducing a new procedure. According to Danish law, no additional approval from ethical research committees was required.

### 2.2 Setting and participants

We included all patients referred to and eligible for in-bore MRGB in the Central Denmark Region from August 2014 until February 2020. The Central Denmark Region has a catchment area of approximately 1 300 000 people.

Referral to in-bore MRGB was based on mpMRI ordered by departments of urology in the Central Denmark Region according to guidelines [12]. In 2014, mpMRI was offered to patients with elevated prostate-specific antigen (PSA) and at least one negative TRUS biopsy. From 2015, mpMRI was also offered as a part of active surveillance (AS). Finally, patients could be referred to mpMRI if they had contraindications to TRUS biopsy, for example, ongoing anticoagulation therapy, and/or were immunosuppressed.

The patients formed a consecutive clinical series. No patients were referred to mpMRI or other MRGB systems than mpMRI or in-bore MRGB at the Department of Radiology, Aarhus University Hospital (AUH), Aarhus, Denmark.

### 2.3 Multiparametric MRI and referral for in-bore MRGB

A 3 Tesla Skyra (Siemens, Erlangen, Germany) scanner at the Department of Radiology, AUH, was used for mpMRI. Multiparametric magnetic resonance images were described according to the various Prostate Imaging Reporting and Data System (PIRADS) guidelines through the years [13], [14], [15]. Lesions were assigned a PIRADS score from 1 to 5. A score of 1 signified that the risk for PC was highly unlikely, and a score of 5 signified that PC was highly likely. If a patient had more than one lesion in the prostate, the lesion with the highest score was considered the index lesion. All clinical information was available to the radiologists at the time of mpMRI.

Interpretation of the mpMRI images (and performance of in-bore MRGB) was done by one of three senior radiologists at the Department of Radiology, AUH, which is a certified Center of Excellence in mpMRI of the prostate. The certification was given by Radboud University Medical Center, Nijmegen, The Netherlands [16]. All patients had MRI scans with adequate diagnostic quality according to PIRADS [13], [14], [15]. Patients with a PIRADS score of 1–2 were not offered MRGB. Patients with a PIRADS score of 3–5 were evaluated for in-bore MRGB at a multidisciplinary team meeting. Referral for in-bore MRGB was based on the patient’s and urologist’s discretion.

### 2.4 In-bore MRGB

**Figure 2:**
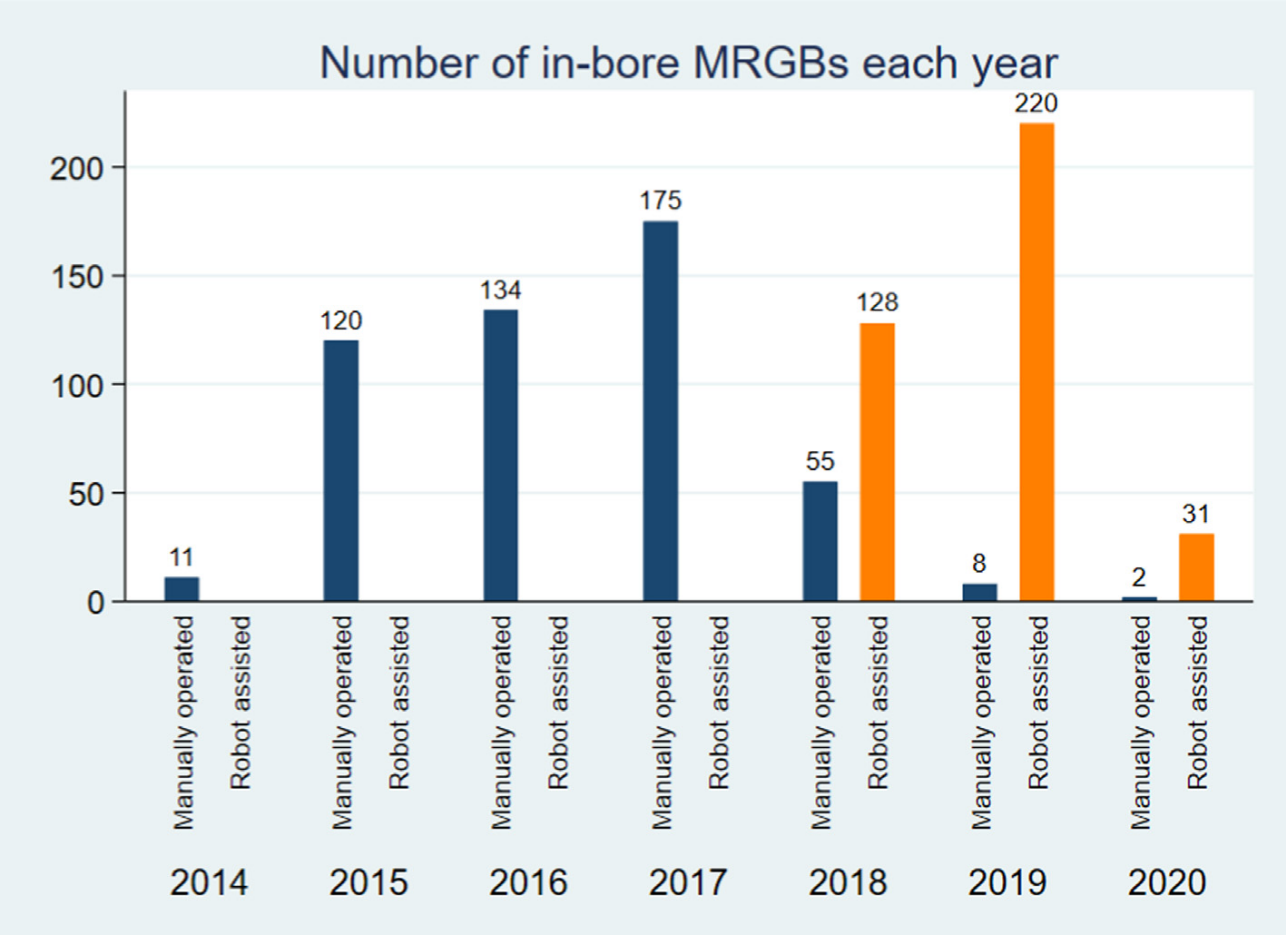
Number of in-bore magnetic resonance imaging targeted prostate biopsies (MRGBs) each year with the manually operated in-bore MRGB system and the robot-assisted in-bore MRGB system. From August 2014 through February 2018, all patients had in-bore MRGB performed with the manually operated system. From March 2018, the in-bore MRGB system of choice was the robot-assisted system and the manually operated system was used only when the robot-assisted was unavailable due to maintenance.

From August 2014 and through February 2018, all patients had in-bore MRGB performed as MO-MRGB (Philips, DynaTRIM; Invivo Corp, Gainesville, FL, USA). From March 2018, RA-MRGB (Soteria Medical, Nijmegen, The Netherlands) was preferred and MO-MRGB was performed only rarely (Fig. 2).

All patients were prepared for in-bore MRGB according to local guidelines containing thorough information about the procedure, antibiotic prophylaxis, local analgesia, and pause of anticoagulation therapy if relevant.

Patients were placed in a 3 Tesla scanner in prone position, and the prostate biopsy was taken transrectally. An MRI-compatible titanium needle (Invivo Corp) was used during MO-MRGB, and the needle position was documented by true fast imaging with steady-state precession in two planes (Supplementary Fig. 1). A steel needle (HistoCore; BIP Medical, Türkenfeld, Germany), which did not allow for real-time MRI scanning, was used during RA-MRGB and the needle position was documented with software (Supplementary Fig. 2). Two or three biopsies were taken per lesion depending on obtained needle position or tissue yield. The planned room time was 60 min for MO-MRGB and 45 min for RA-MRGB.

### 2.5 Histopathology and treatment

Biopsy tissue from each needle was sent as separate samples to the Department of Pathology, AUH, Denmark, and described according to standard clinical practice, that is, Gleason score and International Society of Urological Pathology 2014 grade group [17].

Depending on the biopsy result, patients could receive active treatment, for example, prostatectomy, or be included in AS or watchful waiting (WW). AS and WW included repeated PSA measurements, repeated mpMRI scans, and/or repeated TRUS or MRGB biopsies. Finally, it could be chosen to do nothing further after MRGB.

### 2.6 Outcome measures

The primary outcome was a comparison of the PC DRs in the index lesions based on the in-bore MRGB systems. The secondary outcome was the RD of PC between the in-bore MRGB systems. Clinically significant prostate cancer (csPC) defined as Gleason score ≥7 was also considered for the PC DR.

### 2.7 Statistical analysis

Stata/IC 16.0 (Stata Corporation, College Station, TX, USA) was used for data analysis. We considered a two-sided *p* value of <0.05 as statistically significant.

Median and interquartile range were reported for continuous data, while frequency and proportion were reported for categorical data. Student *t* test was used to compare parametric data, and for nonparametric data, the Wilcoxon Mann–Whitney *U* test was used. Pearson’s chi-square test was used for contingency tables, and Fisher’s exact test was used for contingency tables when the number of any observation was <5.

To account for any changes in population over time, we retrospectively created three binary regression models. Regression model 1 contained only the in-bore MRGB system as a covariate. Regression model 2 consisted of the most clinically important covariates—in-bore MRGB system, age, and PSA. Regression model 3 consisted of all clinically relevant covariates—in-bore MRGB system, age, PSA, prior TRUS, PIRADS score, lesion size, and lowest single slice apparent diffusion coefficient (ADC) <750 μm^2^/s [18]. All *p* values were derived from Wald’s test.

## 3 Results

### 3.1 Descriptive results

**Figure 3:**
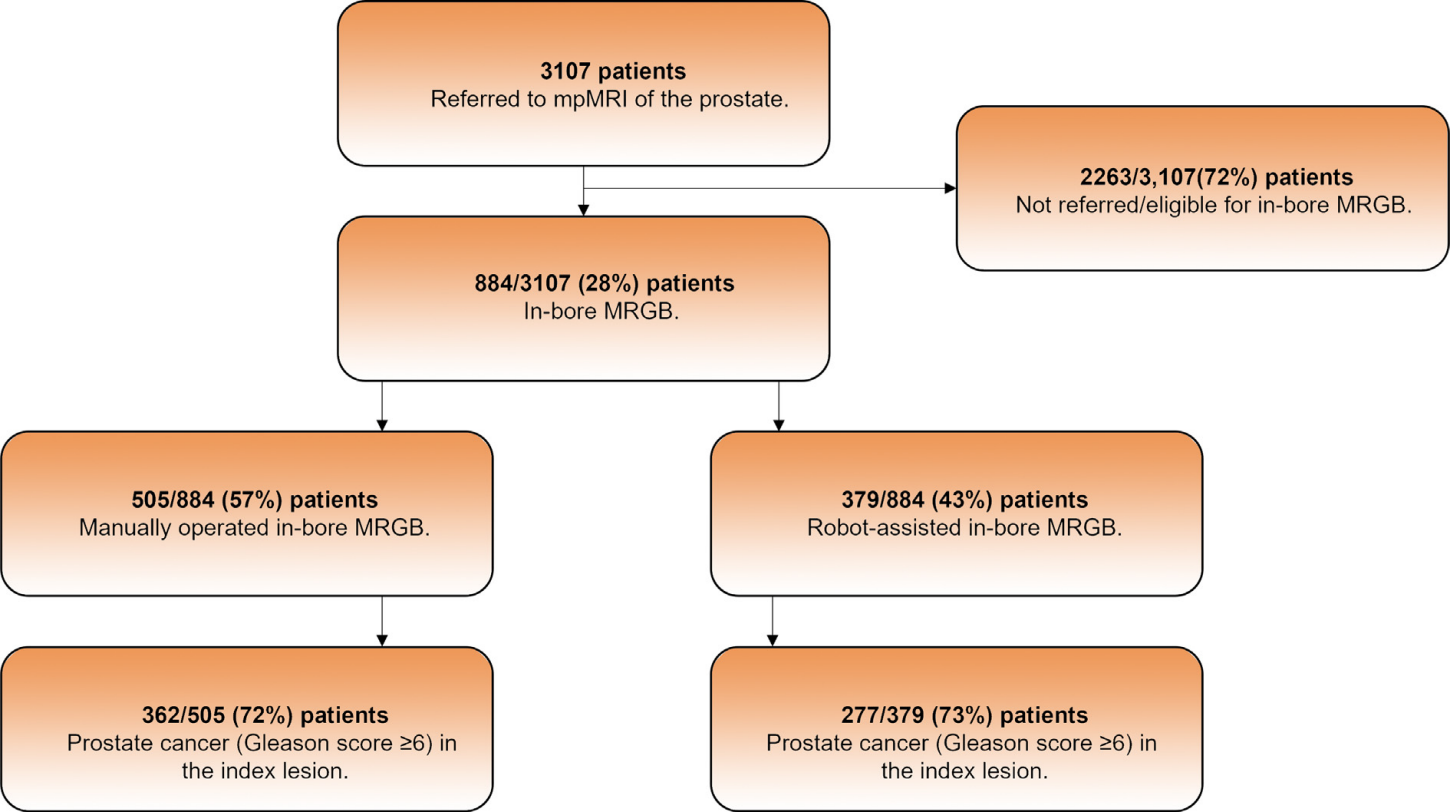
Flowchart of patients in the study. Patients with a PIRADS score of <3 did not receive MRGB. Other exclusion criteria to MRGB were clinical exclusions to mpMRI and/or biopsy such as an unacceptable bleeding risk. The index lesion was considered the lesion with the highest PIRADS score. mpMRI = multiparametric magnetic resonance imaging, MRGB = magnetic resonance imaging targeted prostate biopsy; PIRADS = Prostate Imaging Reporting and Data System.

From August 2014 to February 2020, a total of 3107 patients received mpMRI. Of these, 2263 (72%) patients had a PIRADS score of <3 or were otherwise not referred to or eligible for in-bore MRGB. The remaining 884 (28%) patients, with 1021 PIRADS 3–5 lesions, were referred to in-bore MRGB. The 884 patients were distributed as 505 (57%) in the MO-MRGB group and 379 (43%) in the RA-MRGB group (Table 1 and Fig. 3).

**Table 1:** Baseline characteristics of the patients divided into the manually operated in-bore MRGB group and the robot-assisted in-bore MRGB group

	Manually operated in-bore MRGB (*n* = 505)	Robot-assisted in-bore MRGB (*n* = 379)	*p* value
Age (yr)			0.4^a^
Median	68	67	
Interquartile range	62–71	62–72	
PSA (ng/ml)			<0.001^b^
Median	8.4	7.1	
Interquartile range	6.3–12.0	5.2–10.0	
Prostate volume (ml)			0.6^b^
Median	48	47	
Interquartile range	35–64	36–68	
PSA density (ng/ml/ml)			<0.001^b^
Median	0.18	0.14	
Interquartile range	0.12–0.27	0.10–0.21	
Prior TRUS biopsy, *n* (%)			<0.001^c^
Yes	486 (96)	303 (80)	
Gleason score ≥6, *n* (%)			<0.001^c^
Yes	277 (57)	213 (70)	

MRGB = magnetic resonance imaging targeted prostate biopsy; PSA = prostate-specific antigen; TRUS = transrectal ultrasound guided.

a Student *t* test.

b Wilcoxon Mann–Whitney *U* test.

c Pearson’s chi-square test.

The median age (68 vs 67 yr) and prostate volume (48 vs 47 ml) were not statistically significant (*p* ≥ 0.4) between the MO-MRGB group and the RA-MRGB group (Table 1).

PSA and PSA density were higher (*p* < 0.001) in the MO-MRGB group (median PSA = 8.4 ng/ml and median PSA density = 0.18 ng/ml/ml) than in the RA-MRGB group (median PSA = 7.1 ng/ml and median PSA density = 0.14 ng/ml/ml). The MO-MRGB group had a higher proportion (*p* < 0.001) of prior TRUS biopsies (96%) than the RA-MRGB group (80%). A higher proportion (*p* < 0.001) of patients in the RA-MRGB group had a prior Gleason score of ≥6 than that in the MO-MRGB group (70% vs 57%; Table 1).

### 3.2 Main results

We found no evidence, in our data, of a statistically significant difference (*p* = 0.6) in the DR of PC in the index lesions in the MO-MRGB group compared with that in the RA-MRGB group (72% vs 73%; Table 2). The RD of PC between the in-bore MRGB systems was not statistically significantly different from zero (95% confidence interval: model 1 [–0.05 to 0.07], *p* = 0.6; model 2 [–0.04 to 0.07], *p* = 0.6; and model 3 [–0.03 to 0.08], *p* = 0.4; Table 3).

**Table 2:** Characteristics for the index lesions divided into the manually operated in-bore MRGB group and the robot-assisted in-bore MRGB group^a^

	Manually operated in-bore MRGB (*n* = 505)	Robot-assisted in-bore MRGB (*n* = 379)	*p* value
PIRADS score, *n* (%)			0.061^b^
3	29 (6)	33 (9)	
4	263 (52)	211 (56)	
5	213 (42)	135 (36)	
Mean diameter (mm) on mpMRI			0.6^c^
Median	12	11	
Interquartile range	4–36	5–38	
Longest diameter (mm) on mpMRI			>0.9^c^
Median	15	14	
Interquartile range	5–46	5–46	
Volume (ml) on mpMRI			0.5^c^
Median	0.7	0.6	
Interquartile range	0.2–2.1	0.3–1.9	
Lowest ADC value (μm^2^/s)			>0.9^c^
Median	680	684	
Interquartile range	558–816	548–820	
Location, *n* (%)			0.021^d^
Transition zone	226 (45)	138 (36)	
Peripheral zone	276 (55)	240 (63)	
Central zone	3 (<1)	1 (<1)	
Number of needles, *n* (%)			<0.001^d^
1	4 (<1)	9 (1)	
2	414 (82.0)	352 (93)	
3	87 (17)	18 (5)	
Gleason score, *n* (%)			
≥6			0.6^b^
Yes	362 (72)	277 (73)	
≥7			0.012^b^
Yes	255 (51)	159 (42)	
ISUP grade group >2, *n* (%)			<0.001^b^
Yes	111 (22)	42 (11)	

ADC = apparent diffusion coefficient; ISUP = International Society of Urological Pathology 2014; mpMRI = multiparametric magnetic resonance imaging; MRGB = magnetic resonance imaging targeted prostate biopsy; PIRADS = Prostate Imaging Reporting and Data System.

a The index lesion was the lesion with the highest PIRADS score.

b Pearson’s chi-square test.

c Wilcoxon Mann-Whitney test.

d Fisher’s exact test.

**Table 3:** Regression models^a^

	Risk difference	95% CI	*p* value
Regression model 1—in-bore MRGB system as a predictor for Gleason score ≥6	0.01	–0.05 to 0.07	0.6^b^
Regression model 2—in-bore MRGB system as a predictor for Gleason score ≥6	0.02	–0.04 to 0.07	0.6^b^
Regression model 3—in-bore MRGB system as a predictor for Gleason score ≥6	0.02	–0.03 to 0.08	0.4^b^

ADC = apparent diffusion coefficient; CI = confidence interval; MRGB = magnetic resonance imaging targeted prostate biopsy; PIRADS = Prostate Imaging Reporting and Data System; PSA = prostate-specific antigen; TRUS = transrectal ultrasound guided.

a Regression model 1 included only in-bore MRGB as a covariate. Regression model 2 consisted of the most clinically important covariates—in-bore MRGB system, age, and PSA. Regression model 3 consisted of all clinically relevant covariates—in-bore MRGB system, age, PSA, prior TRUS, PIRADS score, size of the lesion, and lowest ADC <750 μm^2^/s.

b Wald’s test.

The combined DR of PC for MO-MRGB and RA-MRGB in any lesion (ie, not just the index lesion) was 81% (80% vs 82%, *p* = 0.6; Supplementary Table 2) and the DR, between the radiologists who performed in-bore MRGB, showed no statistically significant difference (*p* = 0.6; Supplementary Table 1).

We found relatively more (*p* = 0.021) peripheral zone lesions (63%) in the RA-MRGB group than in the MO-MRGB group (55%) and a corresponding difference in the number of transition zone lesions (Table 2).

The proportion of patients with a Gleason score of ≥7 was higher (*p* = 0.012) in the MO-MRGB group (51%) than in the RA-MRGB group (42%; Table 2).

The number of needles used was most often 2 in the MO-MRGB and RA-MRGB groups, but there was a difference (*p* < 0.001) in the distribution between the groups (Table 2).

We found no statistically significant difference (*p* ≥ 0.061) between the index lesions in the groups regarding the PIRADS score, mean diameter, longest diameter, volume, and lowest ADC value (Table 2).

## 4 Discussion

In this unique prospective single-center study, with a large cohort of 884 patients, we found no statistically significant difference in the DR of PC, when using MO-MRGB compared with RA-MRGB (72% vs 73%; Table 2). To account for differences in population characteristics that arose over time, we made three binary regression models with different covariates and compared the RD between the in-bore MRGB systems, with respect to detecting PC. The type of in-bore MRGB system was not a statistically significant predictor for PC in the regression models (Table 3). Indeed, the RDs of PC between the in-bore MRGB systems were 1–2%. However, the confidence intervals indicate that an RD of up to 8% is possible. When assessing the clinical consequences of missing up to 8% of the lesions, it is important to recognize that in case of benign histology, the clinical follow-up would be planned accordingly—typically a multidisciplinary team decision about a new biopsy attempt or follow-up MRI. If more patients should have been included to demonstrate a small but significant difference, the clinical impact would likely be negligible and other factors such as time consumption and procedure price would be more important.

The DR for PC in the index lesion in the MO-MRGB group of 72% and the DR in the RA-MRGB group index lesion of 73% are similar to DRs reported in other studies [7], [11], [19], [20]. Our overall DR of 81% for PC found in any lesion was also similar to DRs in other studies that mainly used MO-MRGB [7], [8].

This study has many strengths and the most important is our unique cohort from a large catchment area. No other option for MRGB existed in the Central Denmark Region, and therefore, our cohort represented the entire population of interest and not “just” a random and potentially biased subsample. All mpMRI examinations and in-bore MRGB procedures were performed in a single MRI center of excellence by the same three senior radiologists. Furthermore, in Denmark, all citizens have equal rights to health care and no patient would be excluded based on economic grounds.

The shift in patient characteristics is, however, the main limitation in the study. The MO-MRGB group had a greater proportion of prior TRUS biopsies and a higher median PSA density than the RA-MRGB group (Table 1). This indicates that the MO-MRGB patients went through more extensive testing and were on a later disease stage before they were referred to in-bore MRGB. These findings could make it more difficult to perform in-bore MRGB in either the MO-MRGB or the RA-MRGB group. It is possible that the most accessible and largest lesions had already been sampled in the MO-MRGB group, thereby leaving only less accessible and smaller lesions for in-bore MRGB. On the contrary, the MO-MRGB group had a higher median PSA density, which could imply a later disease stage and possibly more visible lesions on mpMRI. The difference of relatively more csPC cases found in the MO-MRGB group, compared with the RA-MRGB group, can probably be explained by the shift in the population characteristics.

However, when we compared the index lesion mean diameter, longest diameter, volume, lowest ADC value, and PIRADS score, there was no statistically significant difference in the index lesions between the MO-MRGB and RA-MRGB groups (Table 2). This supports that the conditions to perform in-bore MRGB in either the MO-MRGB or the RA-MRGB group were comparable.

We found a significant shift in the location of the index lesion from the transition zone to the peripheral zone, when we compared the MO-MRGB group with the RA-MRGB group (Table 2). This difference in location of the index lesion possibly illustrates that TRUS is better at detecting cancer in the peripheral zone than in the transition zone [21]. The different location of the index lesion should nevertheless not cause any problems for the in-bore MRGB accessibility [22].

Another limitation is the increased experience of the radiologist performing MO-MRGB and RA-MRGB. It is possible that the quality of the in-bore MRGB procedures improved over time, but since that would be true for both groups and three different radiologists performed the in-bore MRGB procedures, this is a limitation of minor concern. In fact, in-bore MRGB PC DR has been found not to depend on operator experience [23].

Since access to the prostate through the rectum was the same for MO-MRGB and RA-MRGB, we did not compare infection rates. However, other studies have proved that MRGB is relatively safe [23].

Finally, we did not register the procedure time for either in-bore MRGB group and therefore we cannot conclude on which procedure is faster. However, the planned room time was 15 min shorter for RA-MRGB than for MO-MRGB. Furthermore, the utensils used for RA-MRGB was approximately 400 USD cheaper than the utensils used for MO-MRGB. The acquisition price was similar for the MO-MRGB and RA-MRGB systems.

MRI pathways in PC diagnosis, including different prostate MRGB methods, has been researched extensively and has shown a high DR of PC, few complications to the biopsy procedure, and a lower DR of non-csPC cases [5], [24], [25]. Since 2011/2012, RA-MRGB of the prostate has been a promising biopsy method, not the least because of easier adjustments of the biopsy needle [26], [27]. Later, various studies have found benefits from RA-MRGB, including quickness of the procedure, safety, and DR of PC [8], [11], [19], [20].

This study improves our knowledge about in-bore MRGB. It is relevant to clinical practice because we included all patients referred to in-bore MRGB and thus the exact patient population we wanted to study. Furthermore, this is the first study to compare MO-MRGB with RA-MRGB in a single center. A cost-effectiveness analysis of the in-bore MRGB systems could help select the optimal system.

## 5 Conclusions

In our large cohort, we did not see evidence of an effect on the DR or the RD for PC, when we compared MO-MRGB with RA-MRGB. We cannot completely rule out a small RD, but we find cost-effectiveness considerations more important when choosing the MRGB system.

  ***Author contributions*:** Mads Sandahl had full access to all the data in the study and takes responsibility for the integrity of the data and the accuracy of the data analysis.

*Study concept and design*: Pedersen.

*Acquisition of data*: Pedersen, Marinovskij, Nielsen, Borre, Ulhøi.

*Analysis and interpretation of data*: Pedersen, M. Sandahl, K.J. Sandahl.

*Drafting of the manuscript*: Pedersen, M. Sandahl, K.J. Sandahl, Sørensen.

*Critical revision of the manuscript for important intellectual content*: All authors.

*Statistical analysis*: Pedersen, M. Sandahl, K.J. Sandahl with help from the Department of Biostatistics, Aarhus University, Denmark.

*Obtaining funding*: None.

*Administrative, technical, or material support*: None.

*Supervision*: None.

*Other*: None.

  ***Financial disclosures:*** Mads Sandahl certifies that all conflicts of interest, including specific financial interests and relationships and affiliations relevant to the subject matter or materials discussed in the manuscript (eg, employment/affiliation, grants or funding, consultancies, honoraria, stock ownership or options, expert testimony, royalties, or patents filed, received, or pending), are the following: None.

  ***Funding/Support and role of the sponsor*:** None.

  ***Acknowledgments:*** We would like to thank the Department of Biostatistics, Aarhus University, Denmark, for help with the regressions analysis. We would also like to thank Soteria Medical and Philips for permission to use their images.
