# Supplementary material for: Prostate Cancer Detection Rate of Manually Operated and Robot-assisted In-bore Magnetic Resonance Imaging Targeted Biopsy
Source: Eur Urol Open Sci. 2022 May 28;41:88–94. doi: 10.1016/j.euros.2022.05.002 (PMC9257664; doi:10.1016/j.euros.2022.05.002)
Supplement: Supplementary data 1 [file mmc1.docx]

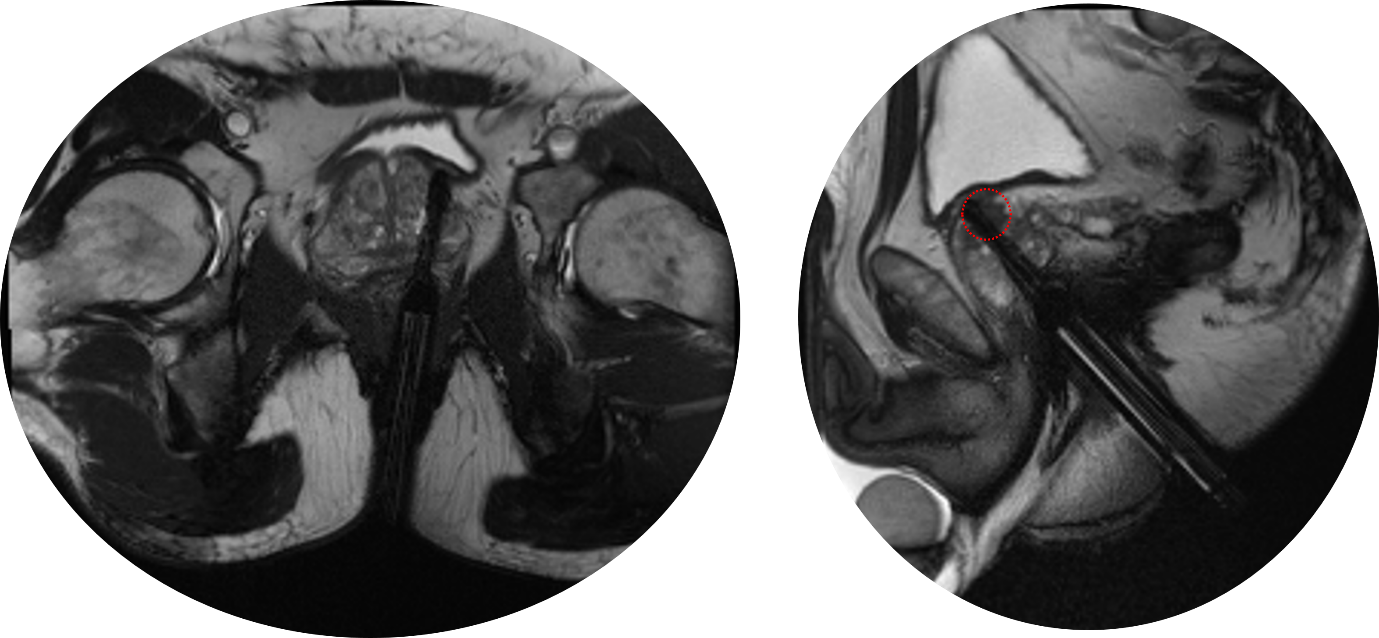


Supplementary Figure 1: An axial (left) and sagittal (right) MRI image taken during MO-MRGB of a 67-year-old man with a PSA = 9.8 ng/ml. The lesion had a PIRADS score of 4 and measured 7*9*9 mm. Due to metal artefact the titanium needle looks rather thick and almost covering the entire dimension of the lesion at axial image. At the sagittal image the lesion is visible (marked with a red circle). Histopathology showed a tumor with a Gleason score of 3+4 in 8/14 mm (57%) of the needle length. **MRI** magnetic resonance imaging, **PIRADS** Prostate Imaging Reporting and Data System, **MO-MRGB** manually operated magnetic resonance imaging targeted prostate biopsy, **PSA** prostate specific antigen.


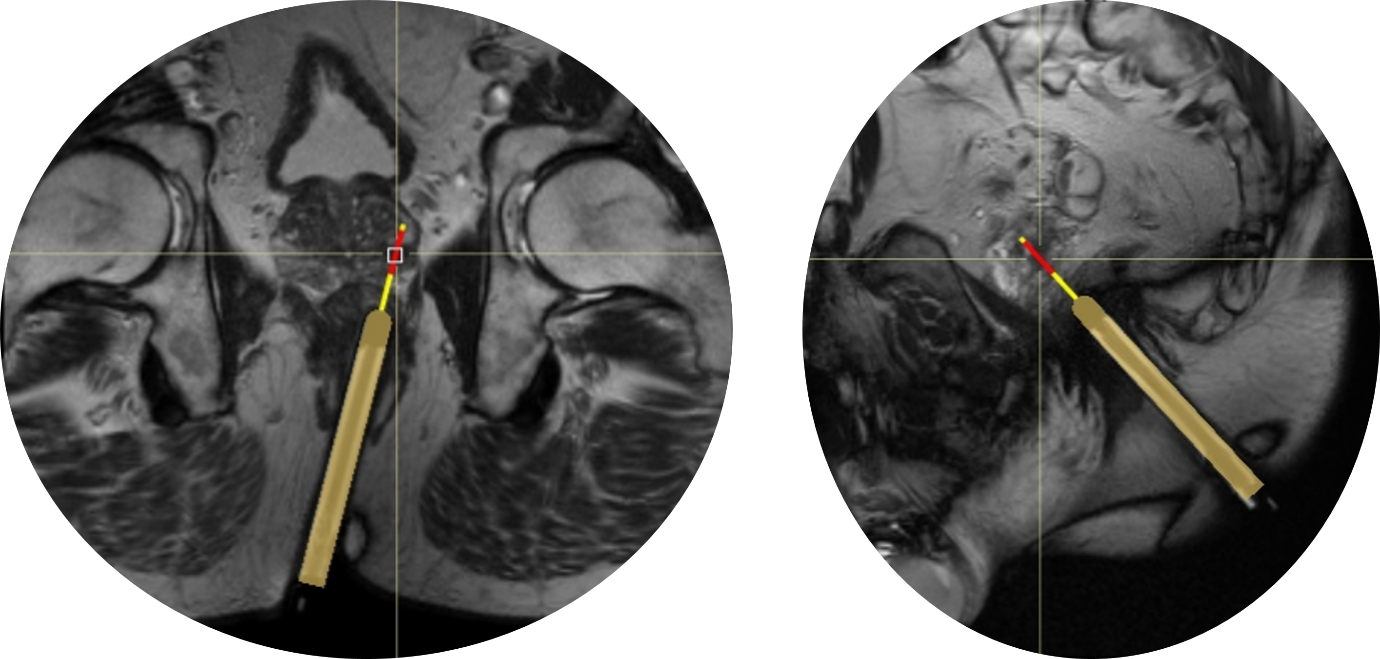


Supplementary Figure 2: An axial (left) and sagittal (right) MRI image taken during RA-MRGB of a 76-year-old man with a PSA = 6.5 ng/ml. The lesion had a PIRADS score of 4 and measured 9*12*12 mm (marked with a yellow crosshair). The needle position is visualized with software documentation. Histopathology showed a tumor with a Gleason score of 3+5 in 4/10 mm (40 %) of the needle length. **MRI** magnetic resonance imaging, **PIRADS** Prostate Imaging Reporting and Data System, **RA-MRGB** robot-assisted magnetic resonance imaging targeted prostate biopsy, **PSA** prostate specific antigen.
