# Supplementary material for: Prostate Cancer Detection Rate of Manually Operated and Robot-assisted In-bore Magnetic Resonance Imaging Targeted Biopsy
Source: Eur Urol Open Sci. 2022 May 28;41:88–94. doi: 10.1016/j.euros.2022.05.002 (PMC9257664; doi:10.1016/j.euros.2022.05.002)
Supplement: Supplementary data 2 [file mmc2.docx]

|  | Mean number of mpMRI scans reported per year from 2016 to 2019, n | Total in-bore MRGBs, n | Total in-bore MRGBs with PC, n |
| --- | --- | --- | --- |
| Radiologist 1 | 314 | 411 | 332 (81%) |
| Radiologist 2 | 200 | 327 | 268 (82%) |
| Radiologist 3 | 204 | 146 | 114 (78%) |
| Total | - | 884 | 714 (81%) |

Supplementary Table 1: Mean number of multiparametric magnetic resonance imaging (**mpMRI)** scans reported from 2016 to 2019 and total numbers of in-bore magnetic resonance imaging targeted prostate biopsies (**MRGBs**) with and without prostate cancer (**PC**) in all lesions performed by each radiologist during the study. The in-bore MRGB detection rates were compared with Pearson’s Chi Squared test and the p value was 0.6.

|  | Manually operated in-bore MRGB (n = 505) | Robot-assisted in-bore MRGB (n = 379) | p value |
| --- | --- | --- | --- |
| PC, n |  |  | 0.6^+^ |
| Yes | 405 (80%) | 309 (82%) |  |

Supplementary Table 2: Overall prostate cancer (**PC**) detection in the manually operated and robot-assisted in-bore magnetic resonance imaging targeted prostate biopsy (**MRGB**) groups. **^+^**Pearson’s Chi Squared test.
